# Somatic cell reprogramming for Parkinson's disease treatment

**DOI:** 10.1002/ibra.12189

**Published:** 2025-01-04

**Authors:** Xiaozhuo Li, Kevin Fang, Fengping Wang

**Affiliations:** ^1^ School of Institute of Primate Translational Medicine Kunming University of Science and Technology Kunming China; ^2^ Living Systems Institute University of Exeter Exeter UK; ^3^ College of Traditional Chinese Medicine Shandong Second Medical University Weifang Shandong China

**Keywords:** cell reprogramming, lineage reprogramming, Parkinson's disease

## Abstract

Parkinson's disease (PD) is a neurodegenerative disease characterized by degeneration of dopamine neurons in the substantia nigra pars compacta. The patient exhibits a series of motor symptoms, such as static tremors, which impair their capacity to take care for themselves in daily life. In the late stage, the patient is unable to walk independently and is bedridden for an extended period of time, reducing their quality of life significantly. So far, treatment methods for PD mainly include drug therapy and deep brain stimulation. Pharmacotherapy is aimed at increasing dopamine (DA) levels; however, the treatment effect is more pronounced in the short term, and there is no benefit in improvement in the overall progression of the disease. In recent years, novel therapeutic strategies have been developed, such as cell reprogramming, trying to generate more DA in PD treatment. This review mainly discusses the advantages, methodology, cell origin, transformation efficiency, and practical application shortcomings of cell reprogramming therapy in PD strategy.

## Graphical abstract

The fundamental purpose of cell reprogramming to treat Parkinson's disease is to generate dopaminergic neurons (DAN) and do transplantation. There are two ways to accomplish this. One method is to induce cells into induced DA neurons (iDAN) directly or to induce cells into induced pluripotent stem cells and ultimately into iDAN in vitro. Another option is in vivo induction. Inject customized astrocytes into a specific brain region to obtain the iDAN.

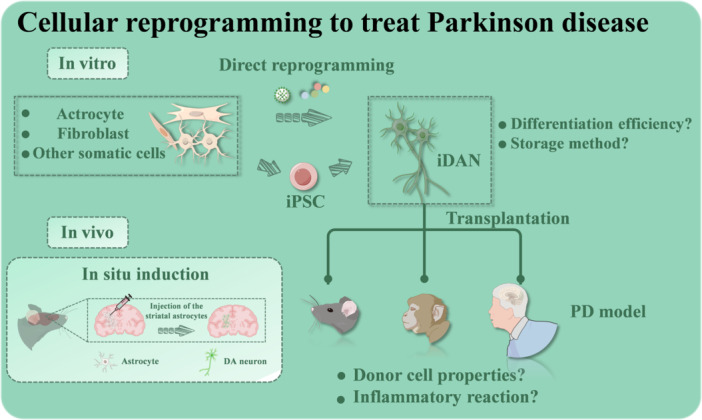

## 1 INTRODUCTION

Parkinson's disease (PD) pathology is characterized by progressive degeneration of dopamine (DA) neurons (DANs) in the dense substantia nigra, and these neurons are involved in the composition of the substantia nigra striatum pathway. This degeneration of striatal DANs leads to reduced DA levels in the striatum and impaired DA signaling in the basal ganglia. Given the role of basal ganglia in a neural circuit for motor regulation and posture maintenance, this disruption leads to clinical symptoms of PD, such as resting tremor, muscle stiffness, bradykinesia, and postural instability.^1^, ^2^, ^3^, ^4^, ^5^ At present, clinical treatment methods for PD mainly include drug therapy (such as levodopa drugs^6^) and electrical stimulation therapy (deep brain stimulation). In recent years, there has also been an electrical stimulation to promote the expression of endogenous neurotrophic factors to reduce the loss of dopaminergic neurons in the substantia nigra.^7^ However, drug treatment and traditional electrotherapy can only temporarily relieve the symptoms of PD and cannot change the disease's progression. As PD advances, the dose of drug therapy needs to be increased, leading to a diminished therapeutic response. Therefore, by regenerating DANs, cell replacement has emerged as a promising therapy for PD.

In initial cell replacement therapy, human fetal ventral midbrain (fVM) tissue was used as a source of DANs.^8^ However, patients receiving these xenografts often experience adverse effects, such as dyskinesia. These complications are attributed to unbalanced DA release from the xenograft and limited ability to reintegrate the target region. At the same time, substitution therapy also faces immune rejection of the xenograft.^9^ The fVM grafts are often mixed with other neuronal types, such as 5‐HTergic neurons, which further complicates the therapy. Due to the low purity of the obtained DAergic neurons, a patient often needs multiple aborted fetuses, posing significant challenges to standardize and apply on a large scale. In addition, different PD progression stages are also important factors in affecting the treatment effect of alternative therapies. Additionally, the ethical issues of tissue origin involved in the treatment continue to pose significant obstacles. In recent years, an emerging human midbrain organoid (hMO) derived from induced pluripotent stem cells (iPSCs) has emerged as a new graft source under 3D culture conditions, which combines the advantages of fVM tissue and two‐dimensional dopamine (2D DA) cells, indicating the potential of hMO as a safe and effective donor graft source for cell therapy for PD.^10^, ^11^

Since 1962, when J. B. Gurdon first succeeded in reprogramming somatic cells into pluripotent stage cells, the landscape of cell reprogramming has undergone significant evolution.^12^, ^13^ This pivotal discovery laid the groundwork for subsequent breakthroughs, for example, Yamanaka first identified the key factors for oocyte reprogramming somatic cells: Oct3/4, Sox2, c‐Myc, and Klf4, marking a true advancement in iPSCs. Additionally, R. L. Davis et al.^14^ found that overexpression of MyoD led to direct reprogramming of fibroblasts into myoblasts, indicating that lineage‐specific transcription factors (TFs) alone are sufficient to alter cell fate. These studies have paved the way for the development of stem cell technology and cell reprogramming, solving the limited source of human DANs, immune rejection, and ethical issues. However, the method of obtaining midbrain‐specific DANs through stem cell induction faces a series of problems, including low post‐transplantation survival rates in vivo, incorrect phenotype of unstable human embryonic stem cells (hESC)‐derived DANs, low cell purification in pre‐transplantation, and post‐transplantation tumorigenic risks arising from undifferentiated pluripotent cells. Moreover, the use of hESC continues to raise ethical issues. Thus, while cell replacement therapy holds promise as a future treatment modality for PD, it requires further refinement and optimization. Due to the high complexity of the pathogenesis of PD and the huge individual differences of patients, the main strategies for the treatment of PD in the future will still include traditional drug treatments and their upgraded and optimized products (such as sustained‐release dosage forms and syringe pumps). However, the emerging field of cell reprogramming therapy warrants deeper exploration as a potentially advantageous strategy. Here, in this review, we will discuss cell reprogramming strategies, factors influencing reprogramming efficiency, and the prospects and drawbacks of this approach for the treatment of PD.

## 2 CELLULAR REPROGRAMMING THERAPEUTIC PD STRATEGIES

### 2.1 Cell preparation and purification

**Figure 1:**
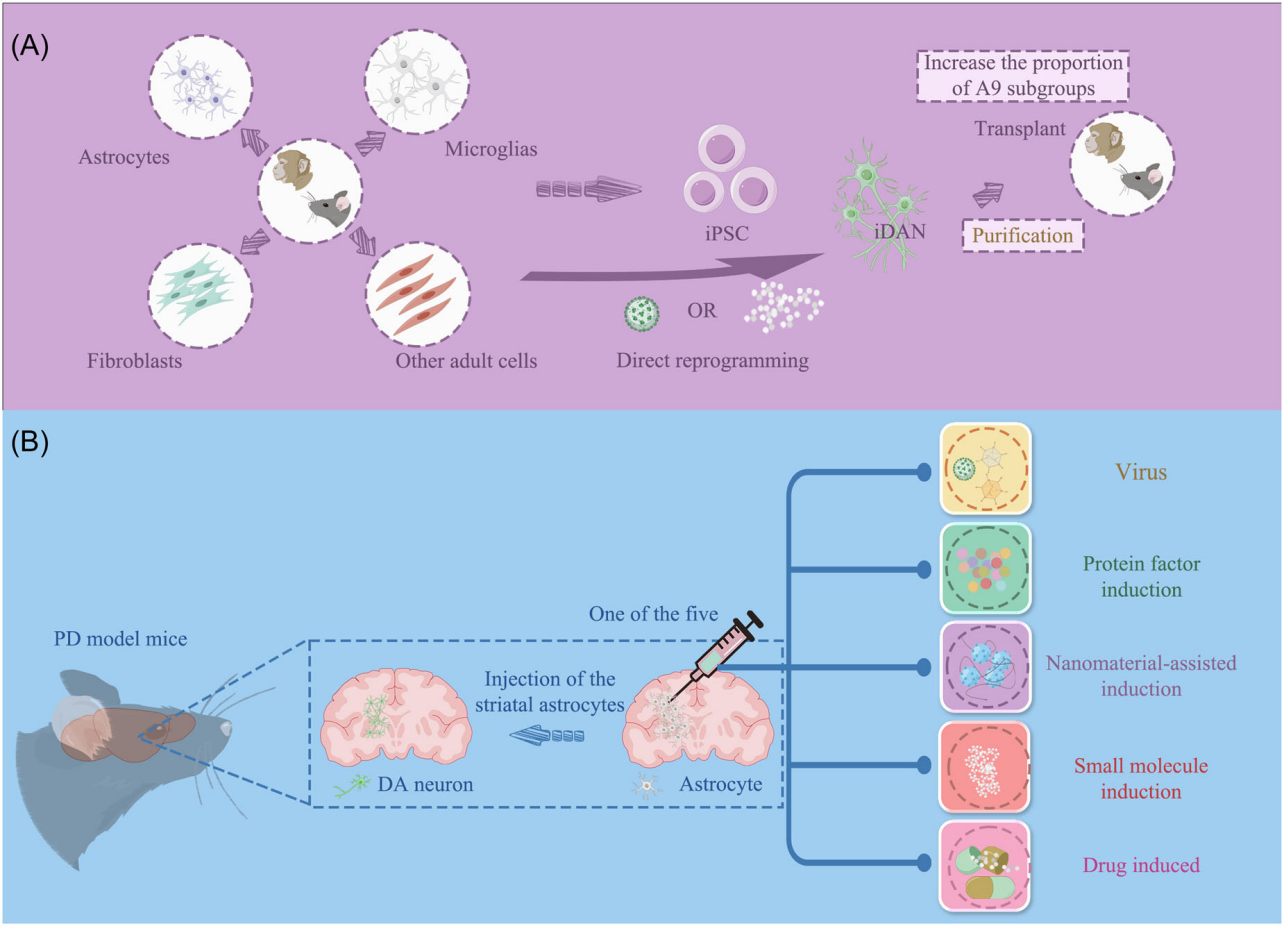
The two major reprogramming strategies. (A) Somatic cells such as astrocytes, microglia, and fibroblasts obtained from monkeys and mice, a type of iPSC obtained by indirect reprogramming and then induced differentiation into dopaminergic neurons, were then transplanted into PD host brains. The other cells are directly reprogrammed into dopaminergic neurons and transplanted them into PD host brains. (B) In the striatum site of the brain of the PD host that enriches the astrocyte population, the orthotopic astrocytes are directly reprogrammed into dopaminergic neurons by viruses, protein molecules, nanomaterials, small molecules, and drug induction. iDANs, induced DA neurons; iPSC, induced pluripotent stem cells; PD, Parkinson's disease. [Color figure can be viewed at wileyonlinelibrary.com]

Cell reprogramming refers to the transformation of cells from one lineage to another. Typically, the strategy is to convert somatic cells into iPSCs by the overexpression of Yamanaka factors: Oct3/4, Sox2, Klf4, and c‐Myc (OSKM). Subsequently, these iPSCs can be differentiated into the cells of interest (Figure 1A). To facilitate the differentiation of iPSCs into neuroectodermal cells, a dual SMAD inhibition approach is employed, involving the BMP antagonist Noggin and TGFβ activator's small molecule inhibitor SB431542, which significantly enhances neural induction efficiency.^15^ Next, dopaminergic mode is initiated in N2 medium using BDNF, ascorbic acid, SHH, FGF8, and maturation is performed in the presence of BDNF, ascorbic acid, GDNF, TGFβ, and cAMP.^16^ P. Stathakos et al. defined a monolayer human iPSC (hiPSC) midbrain dopaminergic (mDA) neuronal differentiation protocol to confirm that FGF8 did not improve the proportion of midbrain cells while increasing GDNF and DAPT concentrations (reaching 20 ng/mL, 10 μM, respectively), leads to a nearly twofold increase in TH‐positive DANs, optimizing the differentiation protocol to rapidly and reproducibly produce a large number of mDA neurons from hiPSCs.^17^ Chanda et al. confirmed that the transcription factor Ascl1 alone is sufficient to induce ESC into DAergic neurons in mice and humans, and the additional Nurr1 and Lmx1a do not increase the proportion of TH+ cells, whereas two neurotrophic factors, BDNF and GDNF, are essential for the successful induction of DANs from ESCs.^18^

The bridge between rodent models and human clinical trials in PD research is testing in nonhuman primates.^19^ Some studies have shown that transplantation of hiPSC‐derived DA precursor cells can restore striatal DA levels and promote functional motor recovery in PD rodent and nonhuman primate models.^20^, ^21^, ^22^, ^23^, ^24^ Following the pioneering work by scientists at Kyoto University,^25^ in Japan, who first implanted iPSC‐derived DA precursor cells into the brains of PD patients in 2018, there has been an increased focus on conducting preclinical studies. These studies aim to evaluate the safety, quality, survival rate, and therapeutic effect of hiPSC/hESC‐derived clinical‐grade DAergic neuronal products.^19^, ^22^, ^26^, ^27^, ^28^ This treatment strategy mainly encounters two primary challenges: one is to ensure the safety of the transplanted cells, and the other is to achieve the long‐term post‐transplantation survival rate, along with the reconstruction of neural circuits in the host brain and the restoration of corresponding functions. Transplantation of incompletely patterned cells carries a risk of tumorigenesis; therefore, enrichment purification before cell transplantation is critical, resulting from the low proportion of DAergic neurons or Dopaminergic neural progenitor cell (DAP) that are suitable for effective transplantation in PD patients.

**Figure 2:**
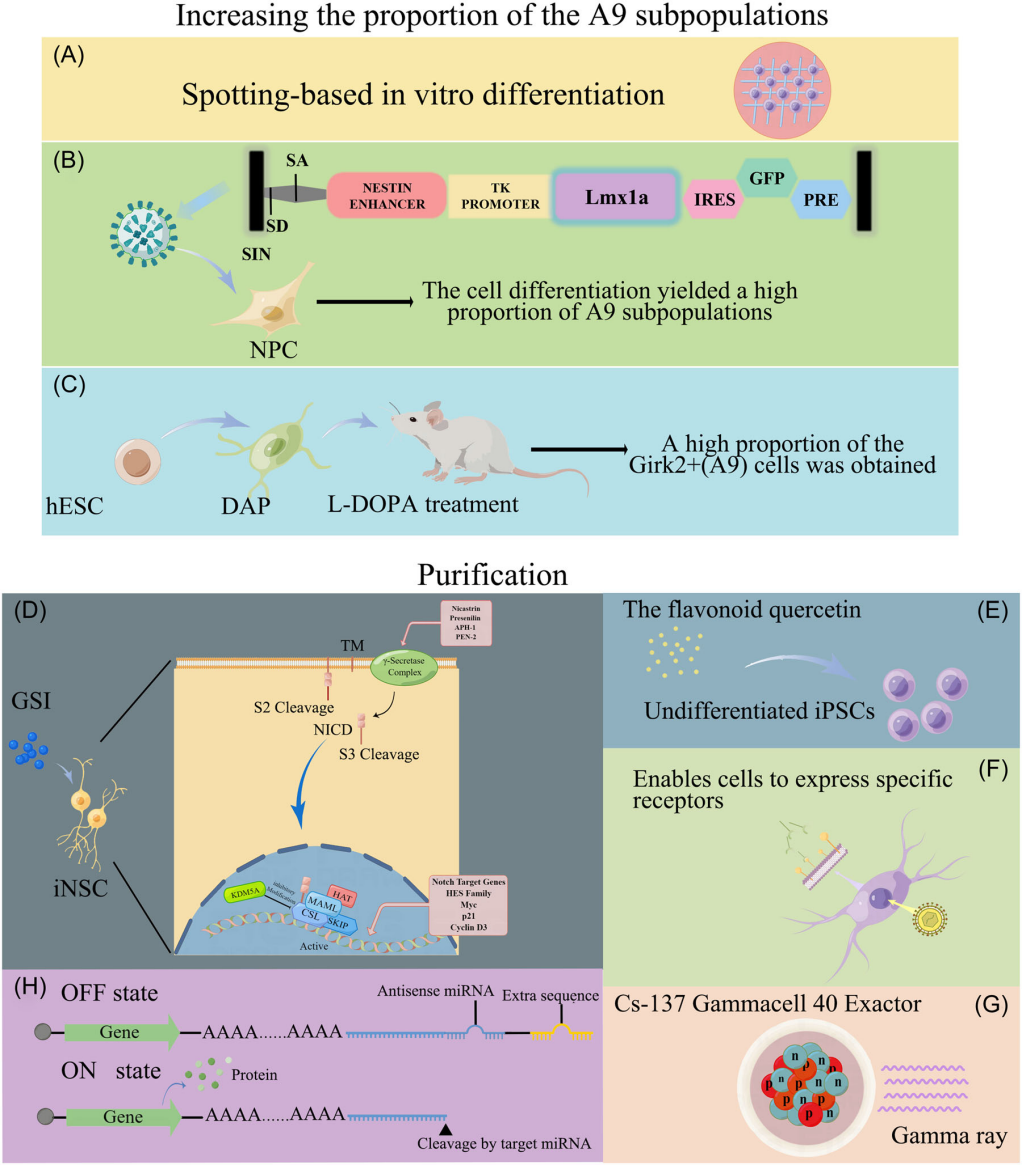
Methods for increasing the proportion of A9 subsets and for purification of the induced cells. (A) Based on optimal physical, chemical, and developmental cues, the researchers developed an efficient and reliable in vitro differentiation method, termed “spotting” method. (B) Lentiviral vectors were used to drive LMX1A overexpression, resulting in the differentiation of LMX1A‐engineered hESCs to obtain dopaminergic neurons with a high proportion of A9. (C) After DAergic neural progenitor cells were transplanted into the brain of PD rats, the proportion of neuron differentiation in the A9 subset was increased by levodopa combined induction. Methods for purification of the induced cells. Small molecules such as γ secretase inhibitors (D), quercetin (E) are used to inhibit Notch signaling in dopaminergic neural progenitor cell, or specific receptors are designed as cell surface markers to selectively eliminate cells (F), or adjuvant radiation therapy after radiation transplantation (G), and cells are purified by RNA switching (H). DAP, dopaminergic neural progenitor cell; hESC, human embryonic stem cell; l‐DOPA, dihydroxyphenylalanine; Girk2+, G‐protein‐activated inward rectifier potassium channel 2; GSI, γ‐secretase inhibitors; iNSC, induced neural stem cell; NPC, neuropithelial cell; NICD, Notch intracellular domain; TM, transmembrane domain. [Color figure can be viewed at wileyonlinelibrary.com]

To address these challenges, two main strategies are employed: the enrichment and selection of the cells of interest, and the elimination or suppression of nontarget cells, especially pluripotent cells. Initially, the researchers used DAergic neuronal surface markers to isolate cells by fluorescence‐activated cell sorting^29^, ^30^, ^31^ or immunomagnetic bead technology. In 2018, Fathi et al. discovered novel markers (GAP43, GSK3β, and VIM) and identified cell surface markers (CNTN2, FLOT2, CALB2, and APMAP proteins) for identifying DAP. These markers can be used for effective cell sorting and purification.^32^ Subsequently, small molecules such as quercetin^21^ and γ‐secretase inhibitors (GSIs), which inhibit Notch signaling in DAP,^33^ along with the incorporation of a suicide gene (cyclinD1‐associated thymidine kinase) into iPSC/hESC lines have been explored.^34^, ^35^ This genetic modification allows for the selective destruction of transduced cells by predrug‐activated gene therapy.^36^ Other methods, including the optimization of iPSC differentiation into mDA using the DNA crosslinker mitomycin‐C (MMC),^37^ post‐transplant adjuvant radiotherapy,^38^ the design of specific receptors as cell surface markers and the selective elimination of specific monoclonal antibodies^39^ have been used to improve the safety and purity of cell transplantation. In 2022, Fujita et al. constructed an efficient and powerful cell purification system. This system incorporates miRNA‐ON and ‐OFF switches encoding Bn (a deadly RNase that induces cell death by degrading RNA) and its inhibitory protein Bs, enabling to control the survival of target cells and achieving a high degree of cell purification (Figure 2D–H).^40^

### 2.2 Preservation of iPSC‐induced DANs

After obtaining the ideal DANs, to facilitate clinical application, it is urgent to have suitable cell preservation methods to make feasible frozen products for better delivery to a large number of patients. Therefore, the questions about how to effectively preserve cells and how to ensure the viability and functional integrity post‐thaw, while retaining pre‐cryopreservation characteristics also arise accordingly. The solution generally starts from two directions: one is to explore the appropriate form of cell during cryopreservation, whether as single‐cell suspension or neuro‐sphere aggregates form; the second is to optimize the cell cryopreservation scheme and system. At the same time, other parameters can be optimized: thaw rate, cell cryopreservation density, freezing volume, vial type (material, thickness), etc.^41^

In this research, Nolbrant et al.^42^ found that ESC‐derived DA progenitor cells, when frozen in single‐cell suspension, had a 60% survival rate in the brain. Cryopreservation, on the other hand, in the form of transplanted cell aggregates or neuro‐spheres, has the advantages of stronger post‐transplantation viability, maintaining the 3D structure of donor cells, and ease of handling. However, cryopreservation of aggregates is undoubtedly more complex and difficult than single‐cell suspensions, so it needs to be combined with optimal cryopreservation methods. After screening six clinical cryoprotectants and exploring six different cooling conditions, Hiramatsu et al. obtained Bambanker hRM cryopreservation medium combined with proton freezer, which is suitable for the cryopreservation of iPSC‐derived DA neurospheres.^41^ After thawing, it shows good viability (including neurite extension ability) and has the same DA‐specific markers, dopamine secretion, and electrophysiological activity as fresh balls. However, it was noted that the maturation of cryo‐spheres in the body is delayed.

### 2.3 Survival of post‐transplantation DANs

The long‐term development, maturation, and survival of the xenograft in vivo should be considered when using in vitro‐induced DANs for PD therapy. Relying solely on short‐term survival assessments may misjudge the actual treatment effect. Brot et al. converted human fibroblasts to hiPSCs by mRNA reprogramming, and then transplanted hiPSC‐derived DANs isotopically into the substantia nigra pars compacta (SNpc) of PD model mice, with a comprehensive 1‐year follow‐up study to observe the DA neuronal development and maturation.^43^ The findings from this longitudinal study showed that although intra‐substantia nigra transplantation repaired the degenerative striatal pathway in the substantia nigra and induced its functional recovery, the number of DANs affected by apoptosis in the graft gradually declined over time. The ratio of grafted proliferating cells is relatively low, and the number of proliferating cells decreases significantly from 1 to 12 months post‐transplantation (1–12 MPT). The researchers found only 1.3% of the proliferating cells at 6 MPT, and all of them belonged to glial cell progenitor cells. The study also showed for the first time that immature neurons are still present in the graft at the 12 MPT period. One of the key factors affecting transplantation efficiency is the differentiation stage of the cells used for transplantation.^34^ Studies have shown that cell transplantation from the late midbrain floor^24^ to the mDA neuroblast stage^34^ or early mDA neurons^42^ can significantly enhance graft development. Such stages are more conducive to maintaining innervation‐related DA targets, increasing innervation, and facilitating the recovery of motor defects. This suggests that selecting the appropriate developmental stage of DANs for transplantation is crucial for maximizing the therapeutic potential and long‐term viability of the graft in PD treatment strategies.

### 2.4 Mature differentiation of DANs

Research has also shown that ablation of host striatal neurons led to reduced survival of DANs within the graft.^44^ This finding was consistent with the results by Brot et al., showing that due to changes in the dopamine fiber pathway from the striatum to the cortical target pathway, the number of A9 dopamine neuronal subsets (subsets critical for restoring motor function) decreased. Simultaneously, the A10 subpopulation increased, and a significant proportion of transplanted DANs failed to mature into any specific subtype. This shows that in addition to paying attention to the long‐term survival of the graft in the host, we should also pay attention to whether the DANs developed in vivo are mature into the A9 subpopulation that contributes more to the recovery of PD motor symptoms. Based on optimal physical, chemical, and developmental cues, the researchers developed an efficient and reliable in vitro differentiation method, termed “spotting” method (Figure 2A–C). This method enables mDA neurons, generated by hiPSCs differentiation, exhibiting typical gene expression and electrophysiological characteristics of A9 mDA neuronal subsets,^21^, ^34^, ^45^ and enables the transplantation into the PD rat striatum of 6‐Hydroxydopamine hydrochloride (6‐OHDA) lesions salvaging motor defects.

The A9 subtype of ventral midbrain DANs is the main cell population for PD loss, and researchers continue to explore how to increase its proportion in hESC/iPSC‐directed differentiation of DANs. Lmx1a encodes TFs that are critical for ventral midbrain identity, especially in neural progenitor cells. Sanchez‐Danes et al. used lentiviral vectors to drive Lmx1a expression, showing that more than 60% of Lmx1a‐engineered hESC‐producing neurons were A9 subsets, and Lmx1a overexpression also increased the proportion of A9 subsets in hiPSC‐induced DANs (Figure 2B).^46^ Some researchers also found that levodopa combined induction and the transplantation DAergic neuronal progenitor cell grafts into the striatum of PD rat models increased the number of positive cells expressing Girk2 (A9 subpopulation‐specific marker), even though there was no effect on the number of DAergic neurons and fiber growth (Figure 2C).^47^ This emphasizes the necessity of tailored approaches that consider the biological factors influencing post‐transplantation neuronal development.

## 3 DIRECT CELL REPROGRAMMING

Due to the many limitations associated with in vitro cell transplantation, such as cell purity, tumorigenicity, cell preservation, resuscitation activity, and post‐transplantation survival, a critical question arises: whether cells can be reprogrammed in situ into DANs in vivo? Or even directly converted into a subset of A9 neurons? With the emergence of direct cell lineage transformation, the idea of PD treatment has been expanded (Figure 1A).

In 2010, Vierbuchen et al. pioneered the concept of cell reprogramming by identifying a combination of three TFs—Ascl1, Brn2 (also known as Pou3f2), and Myt1l—by screening 19 candidate genes, successfully converting mouse fibroblasts into induced functional neurons (iNs) in vitro.^48^ Direct cell reprogramming, also referred to as direct cell lineage transformation, lineage reprogramming, or trans‐differentiation, indicates the direct transformation of somatic cells into functional neurons, without transitioning through an intermediate pluripotent or pluripotent stem cell state. This method is based on the overexpression of lineage‐specific TFs by transgenic methods. Compared to iPSCs, direct reprogramming is faster and more efficient and has unique advantages in tissue repair, as it enables in situ transformation of cells within the target tissue. This approach overcomes the limitations associated with ex vivo cell manipulation, transplantation, and implantation. Lineage reprogramming strategies offer other significant advantages, such as short cell induction cycles and high trans‐differentiation efficiency. Moreover, this method mitigates ethical issues and reduces the risk of tumorigenesis and immune rejection. Since then, more and more research has focused on direct cell reprogramming in a transgenic way, such as transforming human fibroblasts into DANs^49^ and functional DANs.^50^ Various reprogramming strategies have been employed, including (1) viral delivery of specific cell lineage TF^50^, ^51^, ^52^, ^53^, ^54^, ^55^; (2) small molecules, protein factors, and drug induction^55^, ^56^, ^57^, ^58^; (3) microRNA^59^, ^60^; (4) Nanomaterials to promote transformation (Figure 1B).^61^, ^62^, ^63^

To better understand the history and progress of direct reprogramming, we summarized the research in the trans‐differentiation of adult cells to DANs, shown in Table 1. While significant advances have been made in rodent models, there have been few studies on in situ reprogramming of DANs in nonhuman primates for PD treatment, highlighting the need for further research in this area.

**Table 1:** The trans‐differentiation of adult cells to dopamine neurons by direct reprogramming.

Time	Source cells	Treatment object	Methods	Induced cells	Efficiency	In vitro/vivo	References
2011	Human fibroblasts	NO	Overexpression of Ascl1, Brn2, Myt1l and binding to Lmx1a and FoxA2 expression	iDANs	4%	In vitro	[49]
2011	Human/mouse fibroblasts	NO	Ascl1 (Mash), Nurr1, Lmx1a	iDANs	5%	In vitro	[50]
2011	Mouse fibroblasts	Mouse	Ascl1 and Pitx3 are combined and Lmx1a, Nurr1, Foxa2, and EN1 are added, and SHH and FGF8 are used to promote reprogramming	iDANs precursors	9.1%	In vitro	[64]
2011	Mouse astrocytes	NO	A single polycistronic lentiviral vector delivers three TFs: Ascl1, Lmx1B, and Nurr1	iDANs	18%	In vitro	[55]
2012	IMR90 human fibroblasts	Rat	Ascl1, Ngn2, Sox2, Nurr1, Pitx3	iDANs	1%–2%	In vitro	[52]
2014	Mouse fibroblasts	NO	Oct4, Sox2, Klf4, and c‐Myc were induced 5 days after SHH, FGF8, JAK inhibitor (JI1) and Gsk3β inhibitor (CT99021) were introduced to be combined inhibition	iDANs progenitor cells (First generation iDAP)	iDAPs produce TH+/TUJ1+ DANs 57 ± 7%	In vitro	[65]
2014	Mouse fibroblasts	NO	Transcription factors Ascl1 and Nurr1 are combined with neurotrophic factors (including SHH, FGF8b).	iDANs	33%	In vitro	[66]
2015	Mouse fibroblasts	Mouse	iDAPs is induced by Brn2, Sox2, and Foxa2, then SHH, FGF8 were staged with BDNF, GDNF, IGF1, TGF‐β3, dbc AMP, and ascorbic acid	iDAPs with midbrain identity	iDAPs produce TH+/TUJ1+ DANs 90%	In vitro	[54]
2015	Mouse peripheral blood mononuclear cells Mouse fibroblasts	Mouse	Four TFs—Ascl1, Pitx3, Nurr1, and Lmx1a in combination, combined with the drug doxycycline (dox)‐induced	iDANs	Fibroblasts induce DANs 68%	In vitro	[58]
2015	Human fibroblasts	NO	Ascl1, Nurr1, Lmx1a and miR124, and the transformation efficiency is improved by a combination of p53 inhibition and cell cycle arrest	iDANs	TUJ1+ neurons 31.1 ± 1.9%; TH+ neurons 15.4 ± 1.1%	In vitro	[67]
2016	Mouse fibroblasts	Mouse	Small molecule induction (SB431542, Noggin, RA, bFGF, EGF, GDNF, SHH, FGF8b)	iDANs	68 ± 9.9%	In vitro	[57]
2017	Human astrocytes Mouse astrocytes	Mouse	Three TFs NeuroD1, Ascl1, Lmx1a, and microRNA miR218	iDANs	Human astrocytes 16% (in vitro) Mouse astrocytes 14 ± 8% (in vivo)	In vivo/in vitro	[68]
2018	Human fibroblasts	NO	Nonviral expression vectors include human Sox2, human Pax6, mouse Lmx1a, and/or mouse Foxa2	vmDANs	24.90%	In vitro	[69]
2019	Human fibroblasts	NO	Brn2, Sox2, Foxa2	iDAPs	iDAPs are converted to iDANs TH+/NeuN 96.67%; TH+/MAP2 + 86.75%	In vitro	[53]
2020	GABAergic cortical neurons	NO	Ascl1, Nurr1, Lmx1A	iDANs	20%	In vitro	[70]
2020	Mouse astrocytes Human astrocytes	Mouse	Induction by downregulation of RNA‐binding protein PTB	iDANs	Conversion of mouse astrocytes to DANs TH+/GIRK2+22% (DAN A9 subset) (in vivo)	In vivo/in vitro	[71]
2020	Human fibroblasts	NO	Small molecule and protein factor combinations 6–8 d: VRKFYP + SHH, FGF8b, bFGF, Wnt1, Wnt5a; 7–14 d:KFPL, SHH, FGF9b, bFGF, GNDF, BNDF	DAN‐like cells	>85%	In vitro	[56]
2022	Mouse astrocytes (in vivo) Mouse fibroblasts (in vitro)	Mouse	Lentiviral monocis‐transverson vector delivery of Pitx3, Ascl1, Lmx1a, Nurr1 (APNL), optimized by nanomaterial AuNpR processing	iDANs	≥35% (in vitro) ≥40% (in vivo)	In vivo/in vitro	[72]
2022	Human fibroblasts	NO	shREST, Ascl1, Lmx1a, Lmx1b, Foxa2, Otx2, Nr4a2 combinations	iDANs	TAU+ neurons 70.3 ± 0.3%; TH+ neurons 16.1 ± 2.01%	In vitro	[73]

## 4 SELECTION OF REPROGRAMMED CELL SOURCE

In recent years, researchers have tried to induce or reprogram DANs with a variety of cell sources, such as adult eye progenitor cells,^74^ human spermatogonial stem cells,^75^ human peripheral blood cells,^58^, ^76^ amniotic epithelial cells,^77^ hair follicle stem cells,^78^ chromaffin progenitor cells,^79^ and mesenchymal cells.^80^, ^81^ However, the origin of different iPSCs significantly affects the differentiation ability of iPSCs, affecting their early differentiation stages into DAergic neurons.^82^ Considering the future clinical application, the source cells need to be readily available. Therefore, fibroblasts and peripheral blood mononuclear cells are frequently studied.^43^, ^49^, ^51^, ^53^, ^58^, ^69^, ^73^, ^76^, ^83^

As a cellular source for in situ cell reprogramming therapy in PD, the ideal target should have the primary characteristics of being abundant, widespread presence in the brain, including key regions like the substantia nigra and striatum, and suitable for reprogramming. Astrocytes are undoubtedly the most suitable candidates due to these characteristics.^55^, ^68^, ^71^, ^84^ When the brain has an inflammatory response, a large number of reactive glial cells will appear. Using the reactive astrocytes at the inflammatory site as a source of reprogramming cells could reduce the inflammatory response, increase the level of DANs, shorten the nerve repair process, and avoid secondary damage.^85^ In addition, astrocytes have also been used to promote nerve repair in the brain in elderly PD.^86^ At the same time, the astrocytes in the midbrain themselves have a midbrain identity, potentially requiring fewer TFs for reprogramming into mDA neurons. In 2021, Jiang et al. used clustered reactive astrocytes from infarction of the mouse ischemic stroke model as an endogenous cell source, repairing damaged brain structures while reducing glial scarring.^85^ Since astrocytes are greatly plasticized when they react at the site of injury in the body, in situ astrocytes reprogramming is highly attractive.

## 5 FACTORS INFLUENCING REPROGRAMMING EFFICIENCY

### 5.1 Transcription factors

At present, the main problem with direct reprogramming technology using human cells is its low conversion efficiency. Enhancing the efficiency of human cell reprogramming remains a critical goal. Since reprogramming technology is mainly based on viral delivery of TFs, an initial question is whether conversion efficiency can be improved by incorporating other TFs or optimizing TF combinations. Romanov et al. ^77^ found that using NeAL218 induced a greater number of induced DA neurons (iDANs) compared to the combination of NeuroD1, Ascl1, and Lmx1a. Furthermore, adding miR218 and NeuroD1 to the reprogramming mixture significantly increased the expression of the typical mDA gene by 5 to 20 fold.^68^, ^87^ The efficiency of reprogramming to various cell types is influenced by different TF combinations. In 2022, Giehrl‐Schwab et al. compared several TFs combinations Ascl1, Lmx1a, Nr4a2 (ALN) and Ascl1, Lmx1a, NeuroD1, miRNA218 (ALNe‐218), finding that ALN was more effective in reprogramming astrocytes into GABAergic neurons and the resulting striatal GABAergic neurons were able to alleviate toxin‐induced motor behavior defects.^88^ At the RNA level, Sagal et al. found that the pro‐neural transcription factor Atoh1 acts as a key regulator of neurogenesis, enhancing the efficient conversion of iPSCs into DANs with SHH and FGF8b.^89^ On this basis, Xue et al. established the first mRNA drive strategy in 2019, using the non‐muscle myosin II (NM‐II) complex as a positive regulator of the Atoh1, driving neuronal differentiation of iPSCs into mDA neurons and effectively improving the differentiation efficiency.^90^ Addis et al.^55^ constructed a polycistronic lentiviral vector to efficiently convert astrocytes into functional DA energy neurons to improve the efficiency of cells receiving three TFs‐ Ascl1, Lmx1b, and Nurr1. In summary, the uptake or correct combination of TFs is critical for enhancing the reprogramming efficiency (Figure 1B).

### 5.2 Cell cycle and cell fate

One of the early events in cell reprogramming is a strong acceleration of the cell cycle. This acceleration, occurring in only a small subset of cells, can limit the rate of reprogramming.^91^ Changes in the expression of cyclin and cyclin‐dependent kinases (CDK) were associated with maintaining stem cell pluripotency, self‐renewal, and promoting cell differentiation. It was found that the reprogramming efficiency of human fibroblasts was significantly improved after cyclin B1 or cyclin B1 was co‐expressed with CDK1.^92^ It is well known that p53 is an important target protein for cell cycle check points and regulates cell cycle progression along with its downstream genes and Cyclin‐CDK. Earlier, the link between p53 and its downstream p21 gene and the induction of iPSCs had been identified.^93^, ^94^ Subsequent studies by Jiang et al. and Liu et al. showed that p53 is a key barrier for direct reprogramming of fibroblasts into DANs, and the efficiency of direct reprogramming of human fibroblasts into iDANs can be significantly improved by P53 inhibition.^67^, ^95^

Cell fate and behavior are also essential for reprogramming efficiency. Chromatin, chemical signals, and biophysical factors are the main factors affecting cell fate. Compared with somatic cells, pluripotent stem cells have relatively loose and open chromatin and less heterochromatin. Therefore, somatic reprogramming necessarily involves chromatin remodeling, and heterochromatin loosening is also necessary for the initial stage of reprogramming.^96^, ^97^ In 2017, Rivetti et al. demonstrated that treatment with chromatin remodelers such as valproic acid (VPA) and 5‐aza‐2'‐deoxycytidine (Dec), in conjunction with small molecules that activate TGFβ, SHH, and Wnt signaling pathways, significantly improved the efficiency of astrocytes reprogramming. This treatment also sharply increased the expression of midbrain markers, effectively mimicking midbrain development.^68^ These findings suggest that chromatin remodeling and chemical signals improve the efficiency of cell reprogramming to a certain extent, and can mimic endogenous midbrain DA neuronal development with high quality.

Biophysical factors also play an important role in cell fate, especially the array of complex information contained in the extracellular matrix (ECM), such as mechanical properties and topography, which can trigger cellular responses. The microscale and nanoscale topography of ECM or cell culture substrates is one of the important factors in regulating cell adhesion, proliferation, migration, and differentiation.^61^, ^98^, ^99^, ^100^ As biomaterials become increasingly linked to biomedical undertakings, researchers are attempting to use biomaterials to optimize the cellular microenvironment or nanomaterial particles to assist virus transduction, thereby improving transduction efficiency.^101^ With the help of biomaterials as cell fate transformation enhancers, the direct conversion of fibroblasts into iDANs is improved. In the cell reprogramming process, there is a high energy intensity, resulting in excessive oxidative stress. In 2022, Lee et al. applied a modified nanoporous material (AuNpRs) to the direct lineage reprogramming of DAergic neurons to promote the direct transformation of DAergic neurons by improving oxidative stress during fibroblast reprogramming.^72^ The study discovered that the degree to which fibroblasts are transformed into DANs depends on the porosity of AuNpR, which supports Lim et al.'s finding that the nanotopography environment is suitable for direct differentiation and can improve the differentiation efficiency.^63^ In 2015, Yoo et al. demonstrated that nanopatterned substrates can act as effective stimuli for direct reprogramming and play a key role in cell fate changes during direct reprogramming.^61^ Similarly, a study conducted in 2017 found that direct reprogramming of DANs in vivo can be facilitated by utilizing electromagnetic gold nanoparticles (AuNPs) under specific electromagnetic field (EMF) frequency conditions.^62^ AuNPs had been shown can be exposed to EMF to promote the induction of histone acetyltransferase (HAT) Brd2, which acetylates histone H3K27 and leads to chromatin opening (Figure 1B). These findings indicate that the chemical signalings, chromatin remodeling, and biophysical factors are crucial for the reprogramming optimization.

## 6 SUMMARY

This review summarizes the importance, strategies, and affecting factors of cell reprogramming in PD treatment, showing the feasibility of induced DANs in PD patients in theory. There are also clinical data confirming that cell reprogramming is indeed effective. At the same time, there are also clinical data from investigators who have proved that cell therapy for PD has achieved good and long‐lasting therapeutic effects.^24^, ^102^ With the 4th International Congress of Neuroscience in Kyoto, members of GForce‐PD (A Global Effort to Bring Cell‐Based Therapies to PD Patients) are nearing or have completed the good manufacturing practice (GMP) in the cell production process. They plan to finalize comprehensive preclinical efficacy and safety studies within the next 6‐36 months.^102^ These developments suggest that the era of cell therapy for PD is imminent.

Even though with these promising data and progress on cell reprogramming for treating PD, several concerns need to be considered. When patient‐derived cells were used as initiating cells, they retained age characteristics, and the resulting DANs exhibited typical markers of PD pathology, suggesting that donor cell age was a key factor.^24^, ^34^, ^42^, ^103^ On the one hand, researchers can obtain DANs that closely mimic the characteristics of PD patients for disease research. On the other hand, with the progress of neurodegenerative disease in the elderly, brain cells will be accompanied with different degrees of aging and apoptosis. Given that DANs are very sensitive to the apoptosis environment,^104^ it raises questions about the long‐term survival of reprogrammed DANs post‐transformation, and whether the transformed DANs have pathological features in PD patients? Moreover, it is not only the age, but also age‐related changes in epigenetic clock, transcriptome, microRNA, reactive oxygen species, DNA damage, and telomere length, as well as their metabolic profiles and mitochondrial defects, are preserved, adding complexity to the potential efficacy of these therapies.^73^, ^105^, ^106^, ^107^, ^108^

When considering astrocytes as the most suitable candidates for in situ reprogramming, it is crucial to address not only the issue of donor cell age but also pay attention to the specific brain region in which astrocytes should be reprogrammed to relieve PD symptoms. Studies have shown that reprogramming astrocytes in the striatum yield better therapeutic outcomes compared to ectopic transplantation, highlighting the potential benefits of in situ reprogramming.^109^

In conclusion, cell reprogramming is an ideal method to treat PD; however, numerous challenges still need to be addressed in the in situ reprogramming of DANs to treat PD.

## AUTHOR CONTRIBUTIONS

Xiaozhuo Li and Fengping Wang conceptualized this review. Xiaozhuo Li drafted the manuscript, and Kevin Fang revised the manuscript. All the authors have read and approved the final content of this manuscript.

## CONFLICT OF INTEREST STATEMENT

The authors declare no conflicts of interest.

## ETHICS STATEMENT

Not applicable.

## Data Availability

It is not applicable as no date was generated in this review.

## List of Abbreviations

### 1

**Table:**

Abbreviation	Description
Ascl1	Recombinant achaete scute complex like protein1
Atoh1	Atonal homolog 1
AuNpRs	Applied a modified nanoporous material
BBK	Bambanker
BDNF	Brain‐derived neurotrophic factor
bFGF	Basic fibroblast growth factor
BMP	Bone morphogenetic protein
Brn2	POU domain, class 3, transcription factor 2
cAMP	Cyclic Adenosine monophosphate
c‐Myc	Myelocytomatosis viral oncogene homolog
CDK	Cyclin‐dependent kinases
CPAs	Cryoprotectants
DA	Dopamine
DANs	Dopamine neurons
DAP	Dopaminergic neural progenitor cell
dbc AMP	Dibutyryl‐c adenosine monophosphate
Dec	5‐aza‐2’‐deoxycytidine
ECM	Extracellular matrix
EMF	Electromagnetic field
EN1	Engrailed homeobox 1
ESC	Embryonic stem cell
FACS	Fluorescence‐activated cell sorting
FGF8	Fibroblast growth factor 8
Foxa2	Forkhead box A2
fVM	Fetal ventral midbrain
GDNF	Glial cell line‐derived neurotrophic factor
GMP	Good manufacturing practice
HAT	Histone acetyltransferase
hESC	Human embryonic stem cell
hiPSC	Human induced pluripotent stem cell
iDAN	Induced dopamine neurons
iDAPs	Induced dopaminergic neural progenitor cells
iNs	Induced functional neurons
iPSCs	Induced pluripotent stem cells
Klf4	Kruppel‐like factor 4
Lmx1a	LIM homebox transcription factor 1‐alpha
Lmx1b	LIM homebox transcription factor 1‐beta
mDA	Midbrain dopaminergic
miRNA	MicroRNA
MMC	Mitomycin‐C
MyoD	Myogenic differentiation 1, Myod 1
Myt1l	Myelin transcription factor 1‐like
NEUROD1	Neurogenic Differentiation 1
NM‐II	Non‐muscle myosin II
Noggin	Bone morphogenetic protein (BMPs) antagonists
Nurr1	Nuclear receptor related 1
Oct3/4	POU class 5 homebox 1 (POU5F1)
PD	Parkinson's disease
PAGT	Predrug‐activated gene therapy
Pitx3	Paired‐like homeodomain 3
RA	Retinoic acid
Rnase	Ribonuclease
SB431542	Activin/BMP/TGF‐β pathway inhibitor
SHH	Sonic hedgehog
SNpc	Substantia nigra pars compacta
Sox2	Sex determining region Y‐box 2
TFs	Transcription factors
TGFβ	Transforming growth factor‐β
TH	Tyrosine hydroxylase
vmDANs	Ventral midbrain dopamine neurons
VPA	Valproic acid
6‐OHDA	6‐Hydroxydopamine hydrochloride
5‐HT	5‐hydroxytryptamine
